# Adverse Events Following BNT162b2 mRNA COVID-19 Vaccine Immunization among Healthcare Workers in a Tertiary Hospital in Johor, Malaysia

**DOI:** 10.3390/vaccines10040509

**Published:** 2022-03-25

**Authors:** Aie Yen Tan, Chee Tao Chang, Yong King Yu, Yi Xin Low, Najah Fatehah Mohd Razali, Sui Yan Tey, Shaun Wen Huey Lee

**Affiliations:** 1 Pharmacy Department, Hospital Sultan Ismail, Ministry of Health, Johor Bahru 81100, Johor, Malaysia; joanetan6@gmail.com (A.Y.T.); yyk82@hotmail.com (Y.K.Y.); xindylow1996@gmail.com (Y.X.L.); najahrazali08@gmail.com (N.F.M.R.); suiyan1409@gmail.com (S.Y.T.); 2 Clinical Research Centre, Hospital Raja Permaisuri Bainun, Ministry of Health, Ipoh 30450, Perak, Malaysia; 3 School of Pharmacy, Monash University Malaysia, Jalan Lagoon Selatan, Bandar Sunway 47500, Selangor, Malaysia; shaun.lee@monash.edu

**Keywords:** mRNA vaccines, adverse events, immunization, COVID-19, healthcare workers, Malaysia

## Abstract

Background: The severe acute respiratory syndrome coronavirus 2 (SARS-CoV-2), or 2019 coronavirus disease (COVID-19), was declared as pandemic in early 2020. While several studies reported the short-term adverse events (AE) of the mRNA COVID-19 vaccines, medium-term AE have not been extensively evaluated. This study aimed to evaluate the 6-month side effect profiles of the BNT162b2 mRNA vaccine. Methods: This was a descriptive cross-sectional study conducted in a tertiary hospital. Hospital workers who received two doses of the Cominarty (BNT162b2) mRNA vaccine, six months post-vaccination, were invited to participate in this study. All participants completed a self-reported survey assessing AEs occurrence and severity, duration of onset and recovery and if they previously reported these AEs. Results: Of the 670 respondents who completed the survey, 229 (34.2%) experienced at least one AEs, with a total of 937 AEs reported during the 6-month period. After the first dose, the most common reported localized symptoms were pain (n = 106, 27.2%), swelling (n = 38, 9.8%) and erythematous (n = 12, 3.1%) at injection site. Systemic symptoms reported include fatigue (n = 72, 18.5%), fever (n = 55, 14.1%) and headache (n = 46, 11.8%). After the second dose, pain at site of injection (n = 112, 20.4%), swelling (n = 42, 7.7%) and erythematous (n = 14, 2.6%) were among the localized AE reported, while fever (n = 121, 22.1%), fatigue (n = 101, 18.4%) and headache (n = 61, 11.1%) were the most common systemic AE. The proportion of respondents who experienced moderate (first dose: 156 events; second dose: 272 events) and severe (1st dose: 21 events; 2nd dose: 30 events) AEs were higher after the second dose. Most AEs commonly resolved within 1–2 days, and none required hospitalization. No new onset of AE was observed 7 days post-vaccination. A total of 137 (59.8%) participants did not proceed to formal AE reporting. Conclusion: Most of the AEs reported were of mild to moderate intensity and short-term, consistent with those reported in previous studies. No medium-term finding was detected in the survey. AE reporting was not routinely performed, necessitating the attention of health authorities in order to enhance pharmacovigilance.

## 1. Introduction

The emergence of the severe acute respiratory syndrome coronavirus 2 (SARS-CoV-2), or 2019 coronavirus disease (COVID-19), has resulted in more than five million death globally [1]. Common COVID-19 symptoms include fever, dry cough, shortness of breath and fatigue, and are complicated by pneumonia, acute respiratory distress syndrome, multi-organ failures, septic shock and coagulopathy in severe manifestations [2]. Death rate for COVID-19 varies by age, ranging from 0.3 deaths per 1000 cases in patients aged 5 to 17 years to more than 300 fatalities per 1000 cases in patients aged 85 years or older [2]. Hospital workers are one of the groups highly exposed to the coronavirus. This includes both medical staff, such as doctors, nurses, pharmacists and non-medical staff working in the administrative and support arm, including clerks, cleaners and drivers [3]. COVID-19 immunization could reduce the risk of severe COVID-19 and hospitalizations, particularly among those in the high-risk groups, such as older adults and healthcare workers [4,5].

Adverse event (AE) is defined as an unintended or undesirable response due to a drug or chemical compound during its clinical use [6]. As the COVID-19 vaccines were developed using novel technologies, post marketing surveillance is of great importance to detect rare or long-term side effects. Serious AEs may lead to hospitalization, permanent disability, life-threatening conditions or death. Kadali et al. (2021) reported that a minority of vaccinated healthcare workers visited the emergency department and required hospitalization post COVID-19 immunization [7]. Previous studies suggested that the most common AEs due to the COVID-19 vaccines include pain at the injection site, fatigue, fever and muscle pain [7,8,9,10].

Malaysia rolled out the COVID-19 national immunization program in late February 2021, prioritized for all hospital workers. The vaccination program was subsidized by the government for medical and non-medical workers in all public hospitals. While several studies reported the efficacy and short-term AEs of the mRNA COVID-19 vaccines Cominarty (BNT162b2) [9,11], medium-term AEs among hospital workers have not been extensively evaluated. Hence, this study aimed to fill in the gap by investigating the short and medium-term side effect profiles 6 months post-vaccination among hospital workers working in a tertiary hospital upon completion of two vaccine doses, together with their practice of reporting and symptoms management.

## 2. Materials and Methods

This was a self-administered web-based survey conducted among hospital workers in a tertiary hospital with 704 beds in the Johor state of Malaysia, from October to November 2021.

### 2.1. Setting and Study Population

The COVID-19 immunization program for hospital workers was launched in early April 2021, and included 2612 medical staff and 400 non-medical support staff, including administrative staff, security staffs, those working at the food catering services, technology and information unit, cleaning and engineering. Medical and non-medical support staff who completed 2 doses of COVID-19 vaccines (Cominarty) were eligible for the study.

The questionnaire was adapted from several sources [7,8,9,10,11,12] and consisted of five major sections: (i) demographic characteristics, (ii) date of immunization, (iii) solicited AEs (ten items), severity (mild, moderate severe), duration of onset and recovery (days), (iv) unsolicited AEs, severity (mild, moderate severe), duration of onset and recovery (days), (v) treatment seeking behavior (visit healthcare facilities or self-treatment using any type of medication), AE reporting practice and barriers to AEs reporting. Mild AEs do not limit daily activities but cause transient discomfort; moderate AEs are discomforting, affect daily activities and may require self-treatment; while severe AEs cause significant symptoms that restrict daily activities and may require hospitalization.

Face and content validation was performed by a panel of four subject matter experts comprised of an intensive care specialist and pharmacist, an internal medicine specialist and a medical officer in charge of the vaccination administration center. The initial version of the questionnaire was drafted after a meeting between the investigators, and the content was subsequently reviewed by the subject matter experts. The questionnaire was constructed in the national language, Bahasa Malaysia. It was then pre-tested among 10 healthcare staff in the same hospital to ensure clarity and relevance. Minor modifications were made before the tool was finalized for data collection. The study sample size was estimated using the Raosoft sample size calculator (http://www.raosoft.com/samplesize.html, accessed on 20 August 2021). Assuming a study population of 2612 healthcare workers where 50% experienced an AE, with 95% confidence level and 5% margin of error, the minimum sample size required was 336.

The hospital staff were recruited using the convenience sampling method. We included all health staff who could understand Bahasa Malaysia and completed two doses of Comirnaty. Potential respondents were approached at the vaccination station during the third booster dose immunization, 6 months after their second dose. Before answering the questionnaire, all participants were informed about the study and its purpose, followed by an informed consent. If they are willing to participate, they were required to scan the study specific QR code which directed them to a web-based informed consent form, before proceed to answering the questionnaire.

The data analysis was performed using the SPSS version 20.0 software (Armonyx, NY, USA). Descriptive data were expressed as mean ± standard deviation (SD) for normally distributed data, and median (interquartile range) for non-normally distributed data. The relationship between demographic data and AEs of vaccination was analyzed using Chi-square or Fisher‘s exact test. A value of *p* < 0.05 was considered statistically significant.

### 2.2. Ethics Approval

This study was registered in the National Medical Research Registry (NMRR-21-1728-61198) and approved by the Medical Research and Ethics Committee (MREC) [KKM/NIHSEC/P21-1573(12)]. All participants provided informed consent before commencement of study.

## 3. Results

Out of 697 responses received (response rate: 26.7%, study population 2612), 27 responses were excluded as the questionnaire was incomplete, with missing primary outcomes, and 670 were included into the final analysis. Most of the respondents were female (489, 73.8%), aged between 30–39 years old (345, 51.6%) and Malay ethnicity (481, 72.3%). The largest proportion of respondents were staff nurses (248, 37.3%) (Table 1).

About a quarter of the respondents experienced an AE at first dose (146, 21.8%), while 193 (28.8%) experienced an AE after the second dose. However, most of these AEs were generally self-limiting, and did not require any additional medical treatment at health facilities. Most respondents self-treated the symptoms using medication, which increased after using the second dose (74 versus 123, χ^2^ = 77.053, *p* < 0.001). Among 229 respondents who experienced AEs, 137 (59.8%) did not proceed to reporting. The major reasons of non-reporting include perceived mildness of the AEs (121, 88.3%), lack of reporting knowledge (10, 7.3%) and lack of time (4, 2.9%) (Table 2).

**Figure 1:**
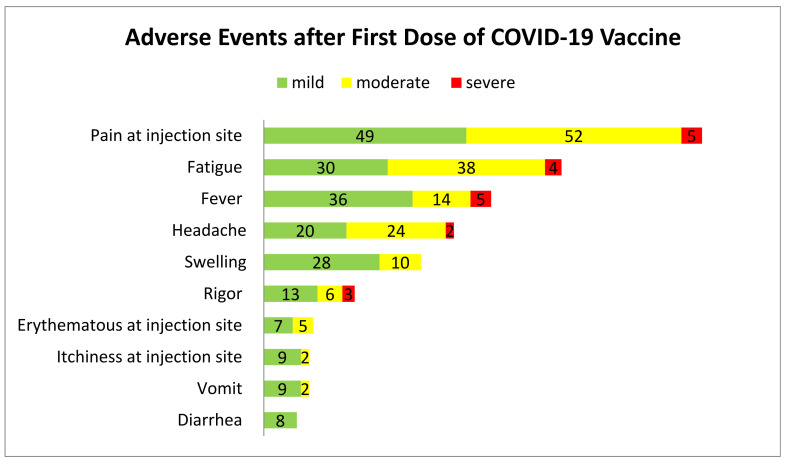
Adverse events after first and second dose of COVID-19 vaccine.

After the first injection, 389 AEs was reported. The most common localized symptoms include pain at injection site (n = 106, 27.2%), swelling (n = 38, 9.8%), erythematous (n = 12, 3.1%); systemic symptoms include fatigue (n = 72, 18.5%), fever (n = 55, 14.1%) and headache (n = 46, 11.8%) (Figure 1).

In the subsequent dose, 548 AEs were reported, which was significantly higher than the first dose (χ^2^ = 197.132, *p* < 0.001). Pain at the injection site (n = 112, 20.4%), swelling (n = 42, 7.7%) and erythematous (n = 14, 2.6%) were among the localized AE reported, while fever (n = 121, 22.1%), fatigue (n = 101, 18.4%) and headache (n = 61, 11.1%) were the most common systemic AEs (Figure 1).

The proportion of respondents who experienced moderate (1st dose: 156 events; 2nd dose: 272 events) level of AEs were higher after the second dose (χ^2^ = 8.331, *p* = 0.003). Generally, the median onset of AEs was at Day 1 and resolved at Day 2. Notably, erythematous at the injection site took 5 days to resolve after the second dose. Overall, the longest AE onset time reported was 7 days (n = 6), while the longest AE recovery time was 7 days post-vaccination (n = 15). No AE was reported after Day 7 of vaccination until the 6th month follow-up.

The most common unsolicited AE was lymph node swelling (n = 9). Of note, 1 respondent developed chest pain at the first dose (moderate severity, onset: Day 5, recovery: Day 5), while 1 experienced palpitation at the second dose (moderate severity, onset: Day 1, recovery: Day 3). Other unsolicited AEs include hypertension, hunger, blur vision, myalgia, shortness of breath, loss of appetite, arm pain, leg swelling, hyperglycaemia, drowsiness and body ache, with one event, respectively. None of the AEs resulted in hospitalization.

To examine if there were predictors of AE occurrence, we examined the relationship between respondent demographics, number of doses of vaccine and AE occurrence. No association between occurrence of AE across gender (χ^2^ = 0.204, *p* = 0.652), age (χ^2^ = 4.255, *p* = 0.235) and ethnicity (χ^2^ = 5.731, *p* = 0.125) were observed.

## 4. Discussion

Overall, about one-third of the respondents who received the Cominarty BNT162b2 vaccine experienced an AE. The incidence and severity of AEs after the second dose were higher in comparison to the first dose. Consistent with previous studies, majority of the AEs were mild or moderate and less than one-tenth required treatment at health facilities [8,10,11,13,14]. While about 5% (51/937) of AE was perceived as severe by the respondents, there was no hospitalization reported. Out of 229 respondents who experienced AEs, three-fifth did not report, mainly due to the perception that the AE was not severe enough to warrant hospitalization. Our study demonstrated that after one week of vaccination, there was no new AE onset and all AE was resolved.

The types of localized AEs reported in this study were comparable to previous reports [7,8,11,13,15]. In particular, there were more local AEs reported after the first dose, compared to the second dose, with the most common local AE being pain at injection site. This was similar to those reported by Menni et al., but their incidence were higher in the second dose (29.2%) compared to the first dose (34.3%) [13]. Only a minority of the respondents rated the pain as severe (9/218, 4.1%) and all reported that it resolved within one week, consistent to other studies [11,15,16]. Nevertheless, we observed a marked difference with two studies among healthcare workers in the United States and Czech Republic, where the AE of pain at injection site was four-fold higher than our study [7,8]. There was a low prevalence (<10%) of other local AE, such as swelling and erythematous after both doses, similar to other studies [11,13].

Meanwhile, the most common systemic side effects were fatigue, fever and headache, in congruence with previous findings [7,8,11,16]. About one-fifth of our respondents reported fever. Likewise, previous studies revealed that the prevalence of fever ranged from 16% to 22% [7,11,16]. This was in contrast to the prospective observational study by Menni et al., where less than 5% of the population experienced fever after both doses [13]. Only a small proportion (13/937, 1.4%) of the respondents rated the fever as severe, similar to those reported by Polack and colleagues [11]. On the other hand, prevalence of headache (11%) and fatigue (18%) in this study were similar to the observation by Menni et al., but markedly lower than several others [7,8,11]. There was also a small number of severe headache (7/937, 0.7%) and severe fatigue (10/937, 1.1%) detected in this study.

In resonance with other studies, we found that systemic side effects were more common and severe in the second dose compared to the first dose [11,13]. Menni and colleagues reported that the total number of systemic side effects was increased from 13.5% to 22% after the second dose, while Ossato and colleagues found that the number of AE reported doubled [13,15]. Polack et al. revealed that more subjects experienced severe fatigue and headache after the second dose [11]. This could be attributed to the T- and B-cell responses to vaccination, where after primed by the first dose, the subsequent dose elicited a higher levels of antibodies and stronger immune response [17].

Furthermore, we did not observe any association between AEs with gender and age. This was in contrast with the previous findings by Ossato and colleagues, where females reported more AE when compared to males [15]. Arguably, Supangat and colleagues found that there was no association between gender and AEs incidence [18]. Evidence suggested that older age was significantly associated with lower immune responses [17]. Hence, lower incidence and severity of systemic reactions was observed in older adults in most studies [8,11,13,15]. However, we did not observe any differences across age, likely due to the relatively large proportion of younger respondents (<50 years old), which did not elicit a difference in the incidence of AEs.

We observed a 13% increment in medication use after the second dose of vaccines. This was similar to those reported by Ossato and colleagues [15]. Similarly, Polack et al. revealed that the number of vaccines recipients who practiced self-medication increased by 17% after the second dose [11]. Nevertheless, the number of respondents who visited health facilities was lower than 10% after both doses. This was in consistence to the findings by Kadali and colleagues, where only 3% required professional medical treatment [7]. No hospitalizations were reported in this study, mainly due to the absence of allergic and anaphylactic reactions among the healthcare workers. It is evident that severe AEs that required hospitalization are rare, and usually resolved without sequela [7,11,15,16].

It is noteworthy that only two-fifths among the respondents proceed to AEs reporting after both doses of vaccines. The majority of those who did not report felt that the AE was not severe enough to warrant reporting, followed by lack of knowledge and time concern. The prevalence of AE reporting among healthcare workers was slightly higher in a study by Gidudu and colleagues, where 55% reported the experienced AE [19], in which 22% provided the similar reason of not reporting. Parrella and colleagues suggested that the lack of knowledge on AE reporting, time constraints and cumbersome reporting process might discourage reporting behavior [20]. Hence, a user-friendly AE reporting platform and supportive organization environment is crucial to increase the intention of AE reporting among healthcare workers.

To the best of our knowledge, this was the first local study that reported AEFI among healthcare workers. A cross-sectional design was conducted at a single time-point; hence, causality could not be determined. As a self-administered questionnaire was used to solicit anonymous self-reported symptoms among the healthcare workers, fidelity of the reported symptoms could not be confirmed. While we achieved the minimal sample size, the survey response rate was relatively low, where the results may not be generalizable to the total population under study due to non-response bias. While the 6-month follow-up gap after the first dose of the vaccine may constitute recall bias, this study elucidated the short and medium-term safety of the vaccines, which was not reported in previous studies. Immunogenicity tests performed in future studies could validate the causality between the AEs and vaccines. Further evaluation of the BNT162b2 mRNA COVID-19 vaccine booster dose AE profile is warranted.

## 5. Conclusions

Short-term AEs after both doses of vaccines were modest in frequency and low in intensity, but more common after the second dose. No medium-term finding was detected in the survey. Both local and systemic symptoms resolved in 1–2 days after the injection, supporting results from previous trials. No difference in AE prevalence was observed between different age groups, gender and ethnicity. No hospitalization was reported within the 6-month post-vaccination follow-up, demonstrating the medium-term safety of the vaccine. AE reporting was not commonly practiced, warranting attention from health authorities to optimize pharmacovigilance.

## Figures and Tables

**Table 1:** Demographic characteristics of COVID-19 vaccines recipients (n = 670).

Characteristics	N	%
Age (mean ± SD), years	34.15 ± 6.81	
Age group, n		
20–29	190	28.4
30–39	345	51.6
40–49	115	17.2
50–59	19	2.8
Gender, n		
Male	174	26.2
Female	489	73.8
Ethnicity, n		
Malay	481	72.3
Chinese	103	15.5
Indian	49	7.4
Others	32	4.8
Occupation, n		
Physicians	175	26.3
Dentists	10	1.5
Pharmacists	34	5.0
Staff nurse	248	37.3
Medical assistant	37	5.5
Pharmacy assistant	6	0.9
Other medical personnel	58	8.8
Non-medical support staff	98	14.7

Missing data: age (n = 1), gender (n = 7), ethnicity (n = 5) and occupation (n = 4).

**Table 2:** Adverse events, management and reporting (n = 670).

	N	%
Experienced an adverse event(s), n		
Yes	229	34.2
No	441	65.8
Experienced adverse events after the first dose, n		
Yes	146	21.8
No	524	78.2
Obtained treatment for adverse events at first dose, n		
Received treatment at health facilities	10	6.8
Self-treatment	74	50.7
None	62	42.5
Experienced adverse events at second dose, n		
Yes	193	28.8
No	477	71.2
Obtained treatment for adverse events at second dose, n		
Received treatment at health facilities	17	8.8
Self-treatment	123	63.7
None	53	27.5
Reported the adverse event, n		
Yes	92	40.2
No	137	59.8
Method to report adverse events, n		
MySejahtera mobile application	78	84.8
Pharmacy department	7	7.6
Others	7	7.6
Reasons for not reporting adverse events, n		
Did not feel necessary to report due to mild AE	121	88.3
Did not know the channel to report AE	10	7.3
Lack of time	4	2.9
Others	2	1.5

## Data Availability

The dataset used in this study can be obtained from the authors upon reasonable requests.
